# A Blood–Bone–Tooth Model for Age Prediction in Forensic Contexts

**DOI:** 10.3390/biology10121312

**Published:** 2021-12-10

**Authors:** Helena Correia Dias, Licínio Manco, Francisco Corte Real, Eugénia Cunha

**Affiliations:** 1 Research Centre for Anthropology and Health (CIAS), Department of Life Sciences, University of Coimbra, 3000-456 Coimbra, Portugal; lmanco@antrop.uc.pt; 2 Centre for Functional Ecology (CEF), Laboratory of Forensic Anthropology, Department of Life Sciences, University of Coimbra, 3000-456 Coimbra, Portugal; eugenia.m.cunha@inmlcf.mj.pt; 3 National Institute of Legal Medicine and Forensic Sciences, 3000-548 Coimbra, Portugal; francisco.m.cortereal@inmlcf.mj.pt; 4 Faculty of Medicine, University of Coimbra, 3000-370 Coimbra, Portugal

**Keywords:** DNA methylation (DNAm), epigenetic age estimation, multi-tissue age prediction models (APMs), Sanger sequencing, SNaPshot

## Abstract

**Simple Summary:**

DNA methylation age estimation is one of the hottest topics in forensic field nowadays. Age estimation can be improved under a multidisciplinary approach, the role of a forensic anthropologist and forensic epigeneticist being crucial in the establishment of new basis for age estimation. The development of epigenetic models for bones and tooth samples is crucial in this way. Moreover, developing models for age estimation using several samples can be a useful tool in forensics. In this study, we built two multi-tissue models for age estimation, combining blood, bones and tooth samples and using two different methodologies. Through the Sanger sequencing methodology, we built a model with seven age-correlated markers and a mean absolute deviation between predicted and chronological ages of 6.06 years. Using the SNaPshot assay, a model with three markers has been developed revealing a mean absolute deviation between predicted and chronological ages of 6.49 years. Our results showed the usefulness of DNA methylation age estimation in forensic contexts and brought new insights into the development of multi-tissue models applied to blood, bones and teeth. In the future, we expected that these procedures can be applied to the Medico-Legal facilities to use DNA methylation in routine practice for age estimation.

**Abstract:**

The development of age prediction models (APMs) focusing on DNA methylation (DNAm) levels has revolutionized the forensic age estimation field. Meanwhile, the predictive ability of multi-tissue models with similar high accuracy needs to be explored. This study aimed to build multi-tissue APMs combining blood, bones and tooth samples, herein named blood–bone–tooth-APM (BBT-APM), using two different methodologies. A total of 185 and 168 bisulfite-converted DNA samples previously addressed by Sanger sequencing and SNaPshot methodologies, respectively, were considered for this study. The relationship between DNAm and age was assessed using simple and multiple linear regression models. Through the Sanger sequencing methodology, we built a BBT-APM with seven CpGs in genes *ELOVL2*, *EDARADD*, *PDE4C*, *FHL2* and *C1orf132*, allowing us to obtain a Mean Absolute Deviation (MAD) between chronological and predicted ages of 6.06 years, explaining 87.8% of the variation in age. Using the SNaPshot assay, we developed a BBT-APM with three CpGs at *ELOVL2, KLF14* and *C1orf132* genes with a MAD of 6.49 years, explaining 84.7% of the variation in age. Our results showed the usefulness of DNAm age in forensic contexts and brought new insights into the development of multi-tissue APMs applied to blood, bone and teeth.

## 1. Introduction

Age estimation is one of the most important issues in forensic contexts. Among the parameters of the biological profile, the estimate of adult’s age at death has always been problematic in forensic anthropology since skeletal aging continues to be largely unknown, and all the available methods continue to fail in the approximation to the real age. In other words, there is a discrepancy between biological and chronological ages; the older, the worse. Despite significant research that has been conducted to face problems of adults’ age at death, there is not a model of age prediction that can be considered very satisfactory. In particular, aging the elderly is lacking age indicators that can discriminate among individuals of seventy, eighty and ninety. Apart from that, the methods that can be applied always depend both on the state of completeness and preservation of the human remains. In forensic anthropology practice, there are many situations where the targeted age indicators are missing and where alternatives are needed. That is the case of some burned remains, dismembered bodies and incomplete bodies, among others. On the other hand, in the case of a fresh body of an unidentified victim, where physiognomic traits are no longer available and with no suspicion of identity, age is always a needed parameter. In those situations, an alternative is also required. Although imaging methods could be a good alternative, we here argue that the genetic approach by means of DNA methylation (DNAm) is also a good choice.

DNAm analysis for age estimation has emerged in the forensic field in recent years. Several age-related markers have been investigated in different tissues, including blood, saliva, buccal swabs, sperm, teeth and bones, allowing the development of tissue-specific age prediction models (APMs) with high accuracy [1]. The development of multi-tissue APMs brought many advantages in forensics, since they can be applied to several contexts with different types of samples. However, the discovery of universal biomarkers of age applied simultaneously to many tissue types can be a challenge, since it has been observed that only a few markers can work well as multi-tissue age predictive markers [2].

To our knowledge, only three reports addressed multi-tissue DNAm age prediction in human individuals. Horvath [3] assessed methylation information of 353 CpGs, developing a highly accurate multi-tissue age predictive model showing a strong correlation between predicted and chronological ages (R = 0.97), and revealing a median absolute difference between chronological and predicted ages of 2.9 years (training set) and 3.6 years (test set). The high accuracy can be explained by the larger number of CpGs included in the model. However, a high number of age markers can also bring a challenge for forensic casework application. Moreover, in the Horvath model a larger error (around 10 years) was observed in several tissues suggesting that the best markers for one tissue may not be the best for another. Using published databases, Alsaleh et al. [4] identified a small set of 10 CpG sites and built a multi-tissue model for blood, semen, saliva, menstrual blood and vaginal secretions with a Mean Absolute Deviation (MAD) from chronological age of 3.8 years. Jung et al. [2] developed a multi-tissue APM applied to blood, buccal swabs and saliva with DNAm captured by a SNaPshot assay using five CpGs located at *ELOVL2, FHL2, C1orf132, KLF14* and *TRIM59* genes. The multi-tissue model showed high accuracy with a MAD from chronological age of 3.553 years. This MAD value was similar to that reported in the same study when developing tissue-specific APMs (MAD = 3.17 years in blood; MAD = 3.82 years in buccal swabs; MAD = 3.29 years in saliva). In addition, Jung and colleagues [2] have observed that the *FHL2* gene is more tissue-specific, revealing strong positive age correlation values in saliva and blood, and a weak age correlation in buccal swabs. They observed also that *ELOVL2* and *TRIM59* seem to work as better multi-tissue markers than *FHL2, C1orf132* or *KLF14*.

Our group previously assessed the methylation information of age-correlated CpG sites in genes *ELOVL2*, *FHL2*, *EDARADD*, *PDE4C*, *C1orf132*, *TRIM59* and *KLF14,* captured by Sanger sequencing and SNaPshot methodologies [5,6,7,8]. Several tissue-specific APMs were developed, including for blood [5,6,7], teeth [8] and bones [8]. Considering the scarcity of multi-tissue APMs developed until now, the present study aimed to re-examine the obtained DNAm levels for these highly age-correlated genes combining the previously addressed tissues to test for a multi-tissue blood–bone–tooth age prediction model (BBT-APM).

## 2. Materials and Methods

### 2.1. Population Sample

A total of 185 samples (76 females, 109 males; aged 1–94 years old) from living and deceased individuals from blood, bones and teeth previously addressed for DNAm levels by Sanger sequencing in genes *ELOVL2* (9 CpGs), *EDARADD* (4 CpGs), *FHL2* (12 CpGs), *PDE4C* (12 CpGs) and *C1orf132* (6 CpGs) [5,6,8], and 168 samples (67 females, 101 males; 1–94 aged years old) from living and deceased individuals previously analyzed using a SNaPshot assay for 5 specific CpG sites in genes *ELOVL2, FHL2, KLF14*, *C1orf132* and *TRIM59* [7,8], were considered for this study. The same samples were addressed in both methodologies; however, some samples failed PCR amplification and were excluded from further analysis, which explains the difference in number between the two methods. The age distribution of each training set was shown in Table S1.

Peripheral blood samples from healthy living individuals of Portuguese ancestry were collected from users of Biobanco—Hospital Pediátrico de Coimbra and other hospitals; blood samples from deceased individuals were collected during routine autopsies, after consulting RENNDA (Registo Nacional de Não Dadores) in Serviço de Patologia Forense da Delegação do Centro do Instituto Nacional de Medicina Legal e Ciências Forenses (INMLCF) and from Bodies Donated to Science (BDS), before the embalming method in Departamento de Anatomia da Faculdade de Medicina da Universidade do Porto (FMUP). Fresh bone samples (rib) were collected, after consulting RENNDA, during autopsy in Serviço de Patologia Forense das Delegações do Centro e Sul do INMLCF. Tooth samples (molars) from living individuals were collected in dentist offices, after written informed consent, and tooth samples from deceased individuals (molars) were collected from BDS in Departamento de Anatomia da FMUP. We excluded individuals with known diseases or other clinical conditions that could influence DNAm levels. All blood and bone samples from dead bodies were collected within five days after death.

The herein developed multi-tissue APM using Sanger sequencing includes: 65 blood samples from healthy individuals (42 females, 23 males; aged 1–94 years old), 68 blood samples from deceased individuals (15 females, 53 males; aged 24–91 years old), 23 tooth samples (15 females, 8 males; aged 26–88 years old) and 29 bone samples (4 females, 25 males; aged 26–81 years old). For the multi-tissue APM developed by SNaPshot, 55 blood samples from healthy individuals (34 females, 21 males; aged 1–94 years old), 59 blood samples from deceased individuals (13 females, 46 males; aged 24–91 years old), 23 tooth samples (15 females, 8 males; aged 26–88 years old) and 31 bone samples (5 females, 26 males; aged 26–81 years old) were considered.

The study protocol was approved by the ethical Committee of Faculdade de Medicina da Universidade de Coimbra (n° 038-CE-2017). For living individuals, written informed consent was previously obtained from adult participants and from children’s parents under the age of 18 years.

### 2.2. Sanger Sequencing of C1orf132 in Blood Samples from Living Individuals

As the *C1orf132* gene was not previously addressed in blood samples from living individuals using the Sanger sequencing methodology, the genomic DNA extracted from blood samples of living individuals using the *QIAamp DNA Mini Kit* (Qiagen, Hilden, Germany) was bisulfite converted using the EZ DNA Methylation-Gold Kit (Zymo Research, Irvine, CA, USA), and submitted to polymerase chain reaction (PCR) amplification using the Qiagen Multiplex PCR kit (Qiagen, Hilden, Germany) for a selected region of *C1orf132*, as previously described [5]. Sequencing was performed in the ABI 3130 sequencer (Applied Biosystems, Foster City) with Big-Dye Terminator v1.1 Cycle Sequencing kit (Applied Biosystems), using primers and conditions previously described [5].

### 2.3. Statistical Analyses

Statistical analyses were performed using IBM SPSS statistics software for Windows, version 24.0 (IBM Corporation, Armonk, NY, USA). Linear regression models were used to analyze the relationships between DNAm levels at CpG sites and chronological age. The simple linear regression coefficients from the highest age-correlated CpGs from each gene for Sanger sequencing data, and from each age-correlated CpG site addressed by SNaPshot, were used to predict the age of individuals in the combined set of blood, bone and tooth samples. For both methodologies, all the statistically significant age-correlated CpG sites were combined for analysis using the stepwise regression approach for selection of the relevant variables to be included in a multi-locus BBT-APM. We calculated the Spearman correlation value, the mean absolute deviation (MAD) and the root mean square error (RMSE) between chronological and predicted ages for the combined training set of samples in both methodologies. For both the training sets, each obtained MAD value was interpreted as either correct or incorrect using a cutoff value according to the standard error (SE) of the estimate calculated for each APM.

In addition, the MAD values were calculated for subsets of four distinct age categories (<30 years, 31–55 years, 56–79 years, >80 years) for each training set used in Sanger sequencing and SNaPshot methodologies.

Validation of the BBT-APMs was performed by 3-fold cross-validation that consists of randomly removing a set of samples from the training set and to develop three independent multiple linear regressions on the remaining samples. Subsequently, each model is used to predict the age of the removed samples assigned as validation sets. An additional validation was performed by splitting the complete data set into two subsets (training and validation sets) and independent regression was calculated for the training set and applied to the validation set. All the independent linear regression equations developed for validation purposes included the same CpG sites that have been selected for development of the final multi-tissue APM for each methodology.

## 3. Results

### 3.1. Multi-Tissue BBT-APM using Sanger Sequencing

DNAm levels of 43 CpGs located at *ELOVL2* (9 CpGs), *EDARADD* (4 CpGs), *FHL2* (12 CpGs), *PDE4C* (12 CpGs) and *C1orf132* (6 CpGs) were assessed in a combined training set of 185 samples, including blood, teeth and bones from Portuguese individuals (76 females, 109 males; aged 1–94 years) using the bisulfite PCR sequencing methodology. The simple linear regression analysis showed that the strongest age-correlated site in each gene was: *ELOVL2* CpG6 (R = 0.759, *p*-value = 6.87 × 10^−36^), explaining 57.3% of the variation in age; *FHL2* CpG1 (R = 0.692, *p*-value = 1.11 × 10^−27^), explaining 47.6% of the variation in age; *EDARADD* CpG3 (R = −0.682, *p*-value = 1.21 × 10^−26^), explaining 46.2% of the variation in age; *C1orf132* CpG1 (R = −0.654, *p*-value = 5.67 × 10^−24^), explaining 42.5% of the variation in age and *PDE4C* CpG2 (R = 0.613, *p*-value = 1.79 × 10^−20^), explaining 37.2% of the variation in age (Table 1 and Supplementary Table S2). A clear positive age correlation was observed for *ELOVL2* CpG6, *PDE4C* CpG2 and *FHL2* CpG1 markers, and a clear negative age correlation was observed for *EDARADD* CpG3 and *C1orf132* CpG1 markers (Supplementary Figure S1). The predicted age of individuals was calculated using the simple linear regression coefficients for the individual strongest age-associated markers allowing us to obtain MAD values of 12.01 years for *ELOVL2* CpG6, 13.23 years for *C1orf132* CpG1, 13.52 years for *EDARADD* CpG3, 13.16 years for *FHL2* CpG1 and 13.58 years for *PDE4C* CpG2 (Table 1).

**Figure 1:**
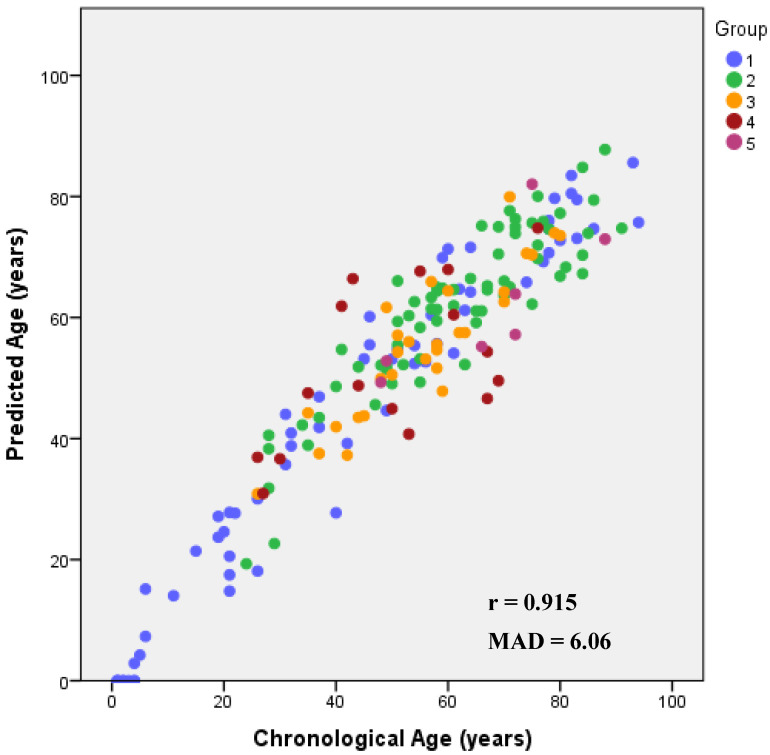
Predicted age versus chronological age using the multi-locus multi-tissue APM developed for *ELOVL2*, *FHL2*, *EDARADD*, *PDE4C* and *C1orf132* genes including blood samples from living individuals (1), blood samples from deceased individuals (2), bone samples (3), tooth samples from living individuals (4) and tooth samples from deceased individuals (5). The corresponding Spearman correlation coefficients (r) are depicted inside each plot.

Simultaneously testing the 35 significant age-associated CpGs from *ELOVL2* (nine CpGs), *EDARADD* (three CpGs)*, FHL2* (nine CpGs), *PDE4C* (eight CpGs) and *C1orf132* (six CpGs) using stepwise regression analysis allowed us to select a multi-locus APM combining seven CpGs (*EDARADD* CpG3, *FHL2* CpG5, *FHL2* CpG11, *ELOVL2* CpG5, *PDE4C* CpG5, *PDE4C* CpG9, *C1orf132* CpG3). The multiple regression analysis combining these CpGs enabled an age correlation (R) value of 0.940 (*p*-value = 7.36 × 10^−79^), explaining 87.8% of the variation in age (corrected R^2^ = 0.878) (Table 1). The formula to predict age of individuals built with the multiple linear regression coefficients (Supplementary Table S3) was as follows: 26.852 − 24.767 × DNAm level *EDARADD* CpG3 + 68.537 × DNAm level *FHL2* CpG5 − 51.319 × DNAm level *FHL2* CpG11 + 57.461 × DNAm level *ELOVL2* CpG5 + 41.449 × DNAm level *PDE4C* CpG5 − 66.397 × DNAm level *PDE4C* CpG9 − 27.418 × DNAm level *C1orf132* CpG3. The correlation between predicted and chronological ages was 0.915 (Spearman correlation coefficient) with a MAD from chronological age of 6.06 years (RMSE = 7.60) (Figure 1). Correct predictions were 73%, assuming that chronological and predicted ages match around eight years, according to the standard error of estimate calculated for the final APM (SE = 7.86).

The accuracy of the developed BBT-APM was tested through a threefold cross validation in the training set of 185 samples showing a MAD of 6.27 years (RMSE = 6.27) (mean value obtained for the three test sets). This value was very close to the MAD of 6.06 (RMSE = 7.60) obtained in the whole training set. The validation by splitting the overall training set into two sets of 117 and 68 samples (training and validation sets) allowed us to obtain an independent MAD value for the training set of 6.09 years (RMSE = 7.55); applying the model on the validation set, a MAD of 6.08 years (RMSE = 7.64) was obtained. Both independent MAD values were very close to the MAD of 6.06 (RMSE = 7.60) obtained in the whole training set.

### 3.2. Multi-Tissue BBT-APM Using SNaPshot Methodology

DNAm levels at five CpG sites from the *ELOVL2, FHL2, KLF14*, *C1orf132* and *TRIM59* genes obtained through a SNaPshot assay were simultaneously addressed in a combined set of 168 samples, including blood, bones and teeth (67 females, 101 males; 1–94 aged years old). DNAm levels of *ELOVL2*, *FHL2*, *KLF14* and *TRIM59* genes revealed a positive correlation with age, and DNAm levels of *C1orf132* showed a negative correlation (Supplementary Figure S2). Testing the individual DNAm association with chronological age for the five CpG sites, the strongest correlation was observed for *ELOVL2* (R = 0.772, *p*-value = 1.54 × 10^−34^), explaining 59.4% of the variation in age, followed by *C1orf132* (R = −0.693, *p*-value = 2.49 × 10^−25^), explaining 47.7% of the variation in age, *FHL2* (R = 0.686, *p*-value = 1.36 × 10^−24^), explaining 46.8% of the variation in age, *KLF14* (R = 0.677, *p*-value = 6.57 × 10^−24^), explaining 45.6% of the variation in age and *TRIM59* (R = 0.584, *p*-value = 1.17 × 10^−16^), explaining 33.7% of the variation in age (Table 2). The simple APMs for each CpG site allowed us to obtain MAD values from a chronological age of 10.95 years for *ELOVL2*, 12.10 years for *C1orf132*, 12.63 years for *FHL2,* 12.74 years for *KLF14* and 13.64 years for *TRIM59* (Table 2).

**Figure 2:**
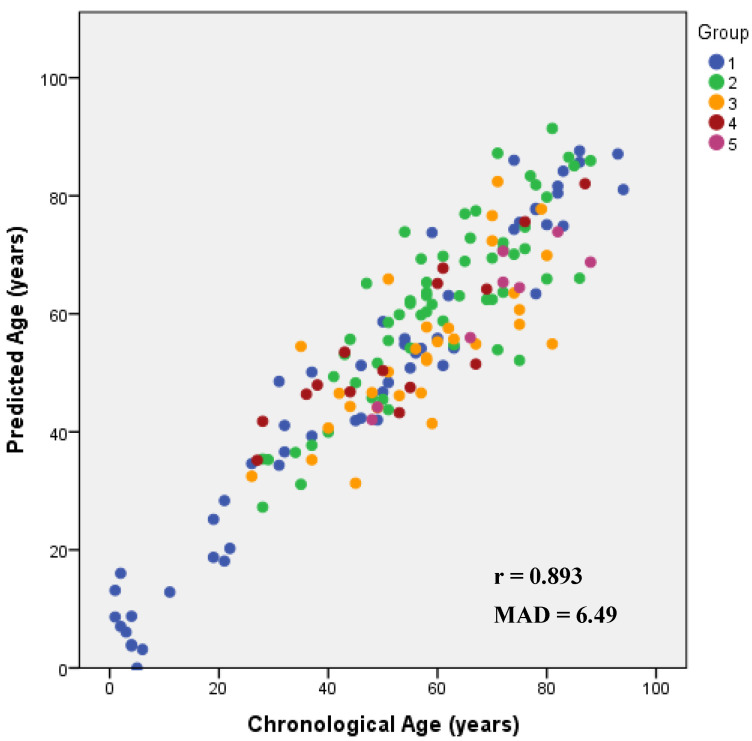
Predicted age versus chronological age using the multi-tissue APM developed for *ELOVL2*, *C1orf132* and *KLF14* genes including blood samples from living individuals (1), blood samples from deceased individuals (2), bone samples (3), tooth samples from living individuals (4) and tooth samples from deceased individuals (5). The corresponding Spearman correlation coefficients (r) are depicted inside each plot.

Applying the stepwise regression approach to the five CpG sites, only the CpGs located at *ELOVL2, KLF14* and *C1orf132* genes were selected for the development of a final multi-locus APM. The three selected CpGs revealed in the multiple regression analysis a very strong correlation with age, R = 0.922 (*p*-value = 3.14 × 10^−67^), explaining 84.7% of the variation in age (corrected R^2^ = 0.847) (Table 2). Predicted age through the multivariate regression coefficients was as follows (Supplementary Table S4): 29.220 + 96.850 × DNAm level *ELOVL2* + 208.747 × DNAm level *KLF14 −* 33.437 × DNAm level *C1orf132.* This BBT-APM allowed us to obtain a MAD from chronological age of 6.49 years (RMSE = 8.42) (Table 2). Correct predictions were 73.8% considering the cutoff of 9 years, according to the standard error of estimate calculated for the final APM (SE = 8.53). The obtained Spearman correlation value between predicted and chronological ages was 0.893 (Figure 2).

The model accuracy of the final APM with DNAm levels of *ELOVL2*, *KLF14* and *C1orf132* markers was evaluated through a threefold cross validation in the training set of 168 samples, producing a MAD (mean value obtained for the three test sets) of 6.73 years (RMSE = 6.75). This value was very close to the MAD of 6.49 (RMSE = 8.42) obtained in the whole training set. The validation by splitting the overall training set into two sets of 113 and 55 samples (training and validation sets) allowed us to obtain an independent MAD value for the training set of 6.06 years (RMSE = 7.81). Applying the model on the validation set, a MAD of 7.45 years (RMSE = 9.60) was obtained.

### 3.3. Differences between Predicted and Chronological Ages with an Increase in Age

Evaluating the model performance obtained with the two developed multi-tissue BBT-APMs according to different age ranges (Table 3), we observed an increase in the MAD values between predicted and chronological ages with the increase in age of individuals. For both Sanger sequencing and SNaPshot methodologies, the value of MAD was the largest for the age group >80 years and the smallest for age group <30 years (Table 3).

## 4. Discussion

In the past decade, several specific epigenetic clocks with high accuracy have been developed using many tissue types [9,10,11,12,13,14,15,16]. However, the discovery of DNAm age-related markers with similarly high accuracy across different types of tissues (universal markers) remains a challenging task in the forensic field [17]. Evidence from previous studies shows that each age-correlated marker reveals a specific ability to predict chronological age, as each tissue type can be affected by different intrinsic or environmental factors. Eipel et al. [16] reported that using a specific APM with methylation information of age-correlated markers selected in one tissue-specific type can lead to a decrease in model accuracy in age prediction if applied to a different tissue. This should be related to the tissue-specific differences in epigenetic patterns [18,19,20]. Thus, a careful selection of age-associated CpGs and the validation of these proposed markers in each tissue type should be the first step for the development of multi-tissue APMs.

In fact, until now, only a few studies have explored the predictive ability of multi-tissue APMs [2,3,4]. In this study, we re-examined DNAm levels of *ELOVL2, FHL2, PDE4C, EDARADD, C1orf132, TRIM59* and *KLF14* genes, previously captured in different tissue types (blood samples from living and deceased individuals; tooth samples from living and deceased individuals; fresh bone samples collected during autopsies) by Sanger sequencing and SNaPshot methodologies to build multi-tissue APMs. We developed simple linear regression APMs for the best-selected CpG sites from each gene, and multi-locus multi-tissues APMs using the best combination of CpGs selected by the stepwise regression approach.

DNAm levels captured by bisulfite Sanger sequencing allowed the development of a final APM with seven CpGs (*EDARADD* CpG3, *FHL2* CpG5, *FHL2* CpG11, *ELOVL2* CpG5, *PDE4C* CpG5, *PDE4C* CpG9, *C1orf132* CpG3), revealing a very strong age correlation value (R = 0.940), highly significant (*p*-value = 7.36 × 10^−79^) and explaining 87.8% of the variation in age. The BBT-APM developed with 185 Portuguese individuals (aged 1–94 years old) allows us to predict age with a moderate accuracy showing a MAD from chronological age of 6.06 years.

Regarding methylation information captured by the SNaPshot methodology, the final multi-locus multi-tissue APM combines three CpG sites located at *ELOVL2, KLF14* and *C1orf132* genes. This BBT-APM developed in 168 samples revealed a very strong age correlation value (R = 0.922), with a MAD from chronological age of 6.49 years.

In Table 4, we resume in brief the difference in results obtained with both methodologies.

The multi-tissue APMs developed herein allows prediction of age of the individuals based on evaluation of DNAm levels captured from several types of samples, including blood, bone and teeth. The final models revealed an accuracy (MAD value) of about 6 years, being more accurate than the majority of anthropological approaches applied to adults’ age estimation. When comparing the results with the ones retrieved by anthropological methods, it becomes clear that our method has clear benefits in relation to methods such as Suchey–Brooks’, where age ranges are particularly large, mainly for old individuals.

Comparing the herein developed multi-tissue APMs with the tissue-specific APMs previously developed by our group, we can observe that through Sanger sequencing, the blood-living APM [6] revealed a MAD of 5.35 years, which is a slightly lower value comparing with the BBT-APM (MAD = 6.06 years). However, for blood samples from deceased individuals [5], the tissue-specific APM revealed a similar accuracy with a MAD of 6.08 years. The tissue-specific APMs developed through the SNaPshot assay for blood samples revealed MAD values of 4.25 and 5.36 years for living and deceased individuals, respectively [7]. However, although these models have a better accuracy than the herein developed BBT-APM using the SNaPshot methodology (MAD = 6.49), they can only be applied to blood samples.

Regarding bones, we have previously obtained through Sanger sequencing and SNaPshot methodologies MAD values of 2.56 and 7.18 years, respectively [8]. Thus, we can observe that for age prediction in bones using Sanger sequencing, it is more advantageous to apply the tissue-specific model compared with the BBT-APM (MAD = 6.06 years). However, using the SNaPshot methodology we obtained a similar prediction accuracy for both the specific bone-APM (MAD = 7.18 years) and the BBT-APM (MAD = 6.49 years). In regards to tooth samples, the tissue-specific models previously developed [8] revealed MAD values of 11.35 years and 7.07 years using Sanger sequencing and SNaPshot methodologies, respectively, which is a lower accuracy in comparison with the BBT-APMs developed in this present study (MAD = 6.06 and 6.49 years, respectively).

Previous reports using DNAm levels for the development of multi-tissues APMs [2,3,4] showed higher prediction accuracy in age estimation (MAD values of 2.9, 3.55 and 3.8 years). In our study, the obtained higher MAD values (6.06 years in Sanger sequencing and 6.49 years in SNaPshot) can be explained by sample size, population variability or the laboratory methodologies for DNAm assessment. Of note, both developed BBT-APMs included CpGs from the *ELOVL2* gene revealing the powerful of this age-associated gene for the development of multi-tissue APMs in forensic contexts. It has been shown that *ELOVL2* is a stable epigenetic marker, revealing a high performance as a multi-tissue predictor [2,13,14,21]. This locus has been used as a powerful age-correlated marker in many tissue-specific APMs developed for blood, tooth, bones and buccal swabs, revealing similar patterns of high accuracy in all APMs [2,10,11,12,13,14,15,22,23,24,25,26,27,28,29,30]. Moreover, it has been shown that CpGs from the other genes addressed in the present study also revealed higher age correlation values in blood samples [2,5,6,7,10,11,12,23,24,26,28,29,30], bones [8,13,14] and tooth samples [8,15,23,27], being promising markers to be selected for development of universal APMs.

Several aspects should be highlighted for future potential applicability of the herein-developed multi-tissues APMs.

In this study, both BBT-APMs revealed a general decrease in model accuracy (increase in MAD value) with the increase in age, in accordance with previous studies [3,11,12,26,30], revealing that age estimation based on DNAm levels can have a better performance in younger age ranges. Indeed, younger individuals show lower values of MAD reflecting a high accuracy in the APMs, comparing to older ages. This reflects larger differences between biological and chronological ages with the increase in age, related to the accumulation of specific alterations in DNAm patterns of each individual with aging due the stochastic or environmental factors, being accepted as the epigenetic drift contribution [31,32,33].

The possibility that postmortem changes can alter the methylation status among specific loci should also be hypothesized, and this issue needs future clarification. As reported in previous studies from our group, comparing blood samples from living and deceased individuals [6,7], it is important for forensic casework application to know the healthy status of the sample donor. This is a paramount issue because the most developed APMs until now have been built using samples of living individuals. It has been observed that ancient DNA (aDNA) can suffer postmortem miscoding lesions, as deamination [34,35]. Postmortem deamination is a spontaneously chemical process that occurs due to the hydrolytic deamination of cytosine (C) residues into uracils (U) [34]. If DNA damage in the form of deamination occurs, the expected residues in PCR amplification could be different after bisulfite conversion. Bisulfite conversion is a chemical modification, which mediates the deamination of unmethylated C to U, appearing after PCR amplification as thymine (T), but leaves methylated C (5mC) intact. Therefore, if postmortem cytosine deamination occurs, both unmethylated C and 5mC appear as T after PCR amplification of bisulfite-converted samples, which could disturb the measurement of DNAm levels. As hydroxymethylcytosine (5hmC) is an oxidative product of demethylation of 5mC [36,37], in case of postmortem deamination, the 5hmC concentration can also be affected. Despite this, the stability of 5mC patterns in aDNA has been reported, when preserved aDNA samples were analyzed [38,39]. Moreover, Pedersen et al. [40] assessed to DNAm levels of permafrost hair samples collected from a Paleo-Eskimo with 4000 years old, and predicted age at death. This reveals the reliability on the assessment of DNAm levels to predict age in ancient samples.

An additional important issue for forensic practice is the effect of postmortem interval (PMI) on DNAm levels captured from aged forensic samples of different tissues. Data obtained from such forensic samples should be interpreted with caution due to the very low amount and degradation of the obtained DNA. A previous study developed by Zbieć-Piekarska et al. [24] showed the stability of prediction accuracy using bloodstains that differed in time of storage. The authors evaluated DNA concentrations from bloodstains that had been deposited previously on tissue paper and kept at room temperature during 5, 10 and 15 years, observing a significant decrease in DNA concentration, a decrease in number of positive PCR amplifications and an increase in average degradation index. However, they did not observe an effect on the rate of corrected predictions, reporting that “the prediction success rate seemed not to correlate inversely with increasing time of storage” [24]. Hence, it seems that DNA degradation affects DNA concentration and, consequently, the rate of positive PCR amplifications; however, the accuracy of age prediction is not affected in positive PCR amplification samples.

The major drawback of our study was the limited number of samples, mainly in bones and teeth. We recognize that larger sample sets have greater statistical power and may be more representative of DNAm changes related to different age groups and different types of tissues, leading to the development of more accurate APMs. Another relevant factor that should be considered is the existence of some diseases or clinical conditions or even some life routines such as smoking or drinking, which may interfere with methylation data. For samples of deceased individuals, despite having access to medical reports of each case, information related to possible clinical conditions was unknown in many cases. Lastly, the use of different methodologies for evaluation of DNAm levels across studies can influence the accuracy of APMs. In particular, bisulfite sequencing or SNaPshot methodologies are semi-quantitative methods and thus may not be the optimal tool for precise DNAm analysis.

DNAm analysis is considered a promising method for age estimation in the future. If we question how easy it is to use it and how long it takes to apply it, we argue that in those laboratories supported by genetic facilities provided with the needed equipment, the results can be retrieved in two or three days. In comparison with the more traditional approaches, it takes longer, but in terms of the delivery of the final report, it does not imply any delay. Furthermore, it should be noted that any method that involves DNA analysis turns out to be more expensive, but it also tends to be more reliable. However, it should be emphasized that the development of universal APMs based on DNAm levels is at the beginning of age estimation research and, therefore, the herein proposed BBT-APMs can be a starting point for future research.

## 5. Conclusions

In conclusion, in this study we re-examined DNAm levels of *ELOVL2, FHL2, PDE4C, EDARADD, C1orf132, TRIM59* and *KLF14* genes previously captured by Sanger sequencing and SNaPshot methodologies across several tissues. Two multi-tissue BBT-APMs were developed using blood, tooth and bone samples from Portuguese individuals. To the best of our knowledge, the two BBT-APMs developed herein for the Portuguese population are the first multi-tissue APMs using bones and teeth. Moreover, despite being very often found in forensic contexts, the development of tissue-specific APMs using bones or teeth is scarce in forensic research. By Sanger sequencing, a moderate accuracy of 6.06 years was obtained in the BBT-APM using seven CpGs from genes *ELOVL2, FHL2, PDE4C, EDARADD* and *C1orf132*. Using the SNaPshot assay, the BBT-APM developed with methylation data from *C1orf132*, *ELOVL2* and *KLF14* genes revealed a MAD from chronological age of 6.49 years. Both methodologies revealed similar accuracy for use in multi-tissue APMs being both simple, rapid, cost-effective and easily available in forensic laboratories. Therefore, both BBT-APMs developed herein can be a promising tool for age estimation in forensic contexts.

This article, a priori, could appear too technical and a little far away from the forensic anthropology reality. However, we argue that a bridge between forensic genetics and forensic anthropology can be achieved, once the needed complicities between the experts involved are well established. In practical terms, what we here advise is an integrated evaluation of the case by the forensic anthropologist, along with the pathologist in charge of the case. If, for instance, the case is a fresh body without any physiognomic traits and where identification is unknown, blood is the best option for DNAm age estimation. If, on the other hand, blood is no longer available due to the state of decomposition of the body, a decision can be made to recover both bone and teeth to perform DNAm studies. What does that imply in practical terms? It means that the result will take 2 or 3 days to be known, that the needed equipment is necessary as well as the adequate kits. While those ones are more expensive than the blood ones, it is a good option in particular when the most adequate skeletal age indicators are damaged or no longer available. Having said that, we argue that we should strive to implement the procedures here described in the Medico–Legal facilities in order to turn DNAm a routine practice for age estimation.

## Figures and Tables

**Table 1:** Simple and multiple linear regression statistics of the age predictors in *ELOVL2, FHL2, EDARADD*, *PDE4C* and *C1orf132* genes to test for association between the DNAm levels and chronological age using Sanger sequencing methodology.

Locus	CpG Site	Location	Multi-Tissue: Type of Samples Included	N	R	R^2^	Corrected R^2^	SE	*p*-Value	MAD
Simple linear regression										
*ELOVL2*	CpG6	Chr6:11044644	Blood * + Bones + Teeth	185	0.759	0.576	0.573	14.70	6.87 × 10^−36^	12.01
*FHL2*	CpG1	Chr2:105399282	Blood * + Bones + Teeth	185	0.692	0.479	0.476	16.29	1.11 × 10^−27^	13.16
*EDARADD*	CpG3	Chr1:236394382	Blood * + Bones + Teeth	185	−0.682	0.465	0.462	16.51	1.21 × 10^−26^	13.52
*C1orf132*	CpG1	Chr1:207823681	Blood * + Bones + Teeth	185	−0.654	0.428	0.425	17.07	5.67 × 10^−24^	13.23
*PDE4C*	CpG2	Chr19:18233133	Blood * + Bones + Teeth	185	0.613	0.376	0.372	17.83	1.79 × 10^−20^	13.58
Multiple linear regression										
APM (*EDARADD* CpG3, *FHL2* CpG5, *FHL2* CpG11, *ELOVL2* CpG5, *PDE4C* CpG5, *PDE4C* CpG9, *C1orf132* CpG3)			Blood * + Bones + Teeth	185	0.940	0.883	0.878	7.86	7.36 × 10^−79^	6.06

Abbreviations: N, number of samples; R, correlation coefficient; SE, standard error; MAD, mean absolute deviation (years) between chronological and predicted ages. Genomic positions were based on the GRCh38/hg38 assembly. * Blood samples from living and deceased individuals.

**Table 2:** Simple and multiple linear regression statistics at the five CpGs of the *ELOVL2, FHL2, KLF14, TRIM59* and *C1orf132* genes to test for association between the DNAm levels and chronological age using SNaPshot assay.

Locus	Location	Multi-Tissue: Type of Samples Included	N	R	R^2^	Corrected R^2^	SE	*p*-Value	MAD
Simple linear regression									
*ELOVL2*	Chr6:11044628	Blood * + Bones + Teeth	168	0.772	0.597	0.594	13.896	1.54 × 10^−34^	10.95
*FHL2*	Chr2:105399282	Blood * + Bones + Teeth	168	0.686	0.471	0.468	15.885	1.36 × 10^−24^	12.63
*KLF14*	Chr7:130734355	Blood * + Bones + Teeth	168	0.677	0.459	0.456	16.091	6.57 × 10^−24^	12.74
*C1orf132*	Chr1:207823681	Blood * + Bones + Teeth	168	−0.693	0.480	0.477	15.779	2.49 × 10^−25^	12.10
*TRIM59*	Chr3:160450189	Blood * + Bones + Teeth	168	0.584	0.341	0.337	17.780	1.17 × 10^−16^	13.64
Multiple linear regression									
APM (*ELOVL2, KLF14* and *C1orf132*)		Blood * + Bones + Teeth	168	0.922	0.850	0.847	8.53	3.14 × 10^−67^	6.49

Abbreviations: N, number of samples; R, correlation coefficient; SE, standard error; MAD, mean absolute deviation (years) between chronological and predicted ages. Genomic positions were based on the GRCh38/hg38 assembly. * Blood samples from living and deceased individuals.

**Table 3:** Evaluation of mean absolute deviation (MAD) between chronological and predicted ages according to four age-range groups in the training set of blood, bone and tooth samples using both methodologies.

	Method	Sanger Sequencing		SNaPshot	
Age Range		N	MAD (Years)	N	MAD (Years)
<30 years		33	4.73	23	5.51
31–55 years		58	6.37	56	6.23
56–79 years		74	5.67	68	6.74
>80 years		20	8.81	21	7.37

**Table 4:** Comparison between Sanger sequencing and SNaPshot methodologies.

Method	Sanger Sequencing	SNaPshot
CpGs and genes included in the APM	7 CpGs located at 5 genes (*EDARADD* CpG3, *FHL2* CpG5, *FHL2* CpG11, *ELOVL2* CpG5, *PDE4C* CpG5, *PDE4C* CpG9, *C1orf132* CpG3)	3 CpGs located at 3 genes (*ELOVL2, KLF14, C1orf132*)
Age correlation value	0.940	0.922
Variance in age explained	87.8%	84.7%
Accuracy (MAD)	6.06 years	6.49 years
Results	Using the Sanger sequencing methodology, more CpGs and genes were included in the APM, but higher age correlation, higher explained variance in age, and a better accuracy in age prediction (lower MAD value) were obtained.	

## Data Availability

Not applicable.

## Supplementary Materials

The following are available online at https://www.mdpi.com/article/10.3390/biology10121312/s1: Figure S1: Correlations between DNAm levels and chronological age in 185 samples including blood samples from living and deceased individuals, bone samples collected from autopsies and teeth from living and deceased individuals, obtained through Sanger sequencing methodology. Figure S2: Correlations between DNAm levels and chronological age in 168 samples, including blood samples from living and deceased individuals, bone samples collected from autopsies and teeth from living and deceased individuals, obtained through SNaPshot methodology. Table S1: Age distribution of the sample sets analyzed by Sanger sequencing and SNaPshot methodologies., Table S2: Univariate linear regression analysis of the 43 CpG sites in *ELOVL2*, *FHL2*, *EDARADD*, *PDE4C* and *C1orf132* loci in 185 samples including blood from living and deceased individuals, teeth from living and deceased individuals and bone collected during autopsies. Table S3: Statistical parameters obtained in a multiple regression model with the seven CpGs in genes *ELOVL2*, *FHL2*, *EDARADD*, *PDE4C* and *C1orf132* selected by stepwise regression approach, in blood, bone and tooth samples. Table S4: Statistical parameters obtained in a multiple regression model with the three CpGs in genes *ELOVL2*, *C1orf132* and *KLF14*, selected by stepwise regression approach, in blood, bone and tooth samples.
