# Supplementary material for: A Blood–Bone–Tooth Model for Age Prediction in Forensic Contexts
Source: Biology (Basel). 2021 Dec 10;10(12):1312. doi: 10.3390/biology10121312 (PMC8698317; doi:10.3390/biology10121312)
Supplement: Supplementary file 1 [file biology-10-01312-s001.zip › biology-1463405-supplementary.pdf]

Supplementary Figures

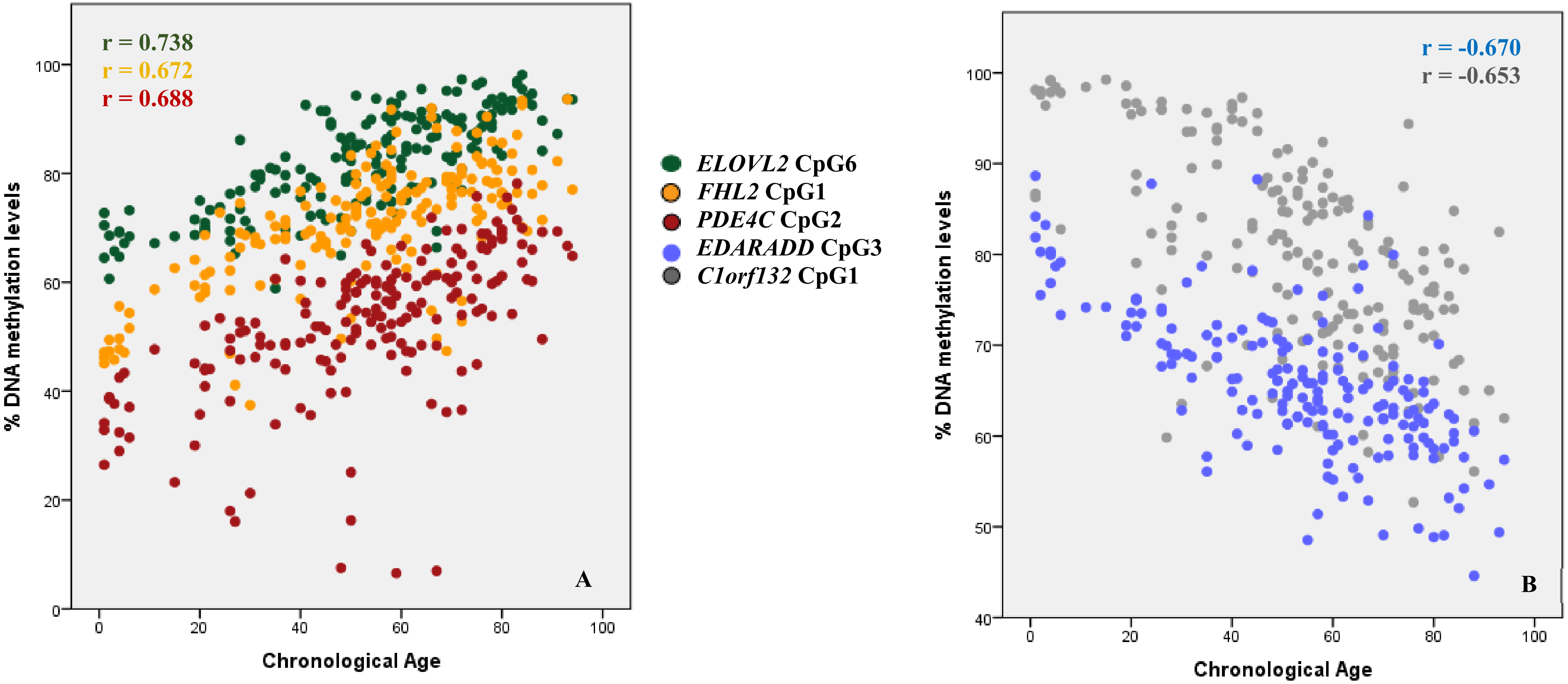

Figure S1: Correlations between DNAm levels and chronological age in 185 samples including blood samples from living and deceased individuals, bone samples collected from autopsies and teeth from living and deceased individuals, obtained through Sanger sequencing methodology. A) Positive correlation between methylation levels and chronological age for *ELOVL2* CpG6 (green), *PDE4C* CpG2 (dark red), *FHL2* CpG1 (yellow) markers; B) negative correlation between methylation levels and chronological age for *EDARADD* CpG3 (blue) and *C1orf132* CpG1 (gray) markers. The corresponding Spearman correlation coefficients (r) are depicted inside each plot.

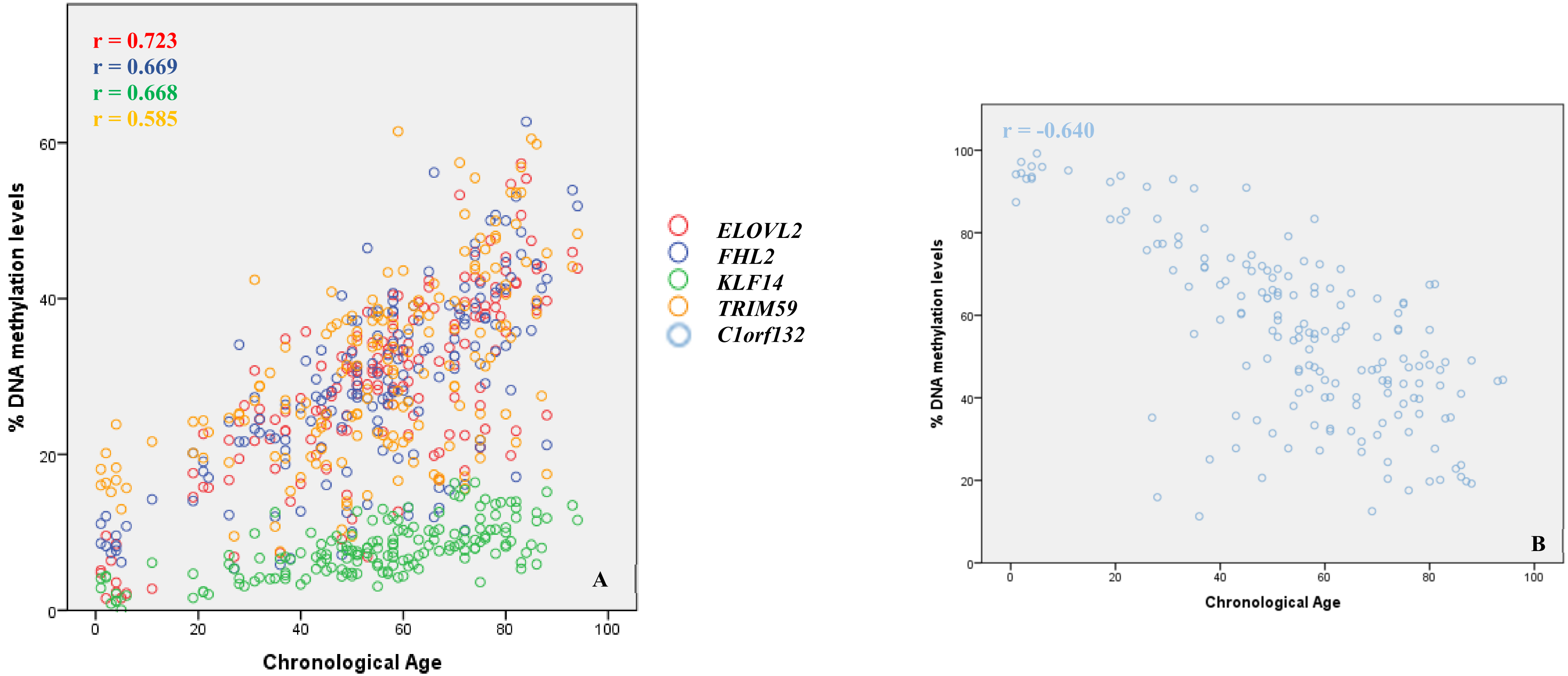

Figure S2: Correlations between DNAm levels and chronological age in 168 samples, including blood samples from living and deceased individuals, bone samples collected from autopsies and teeth from living and deceased individuals, obtained through SNaPshot methodology. A) Positive correlation between methylation levels and chronological age for CpG sites in *ELOVL2* (red), *FHL2* (blue), *KLF14* (green) and *TRIM59* (yellow) genes; B) negative correlation between methylation levels and chronological age for CpG site in *C1orf132* gene (light blue). The corresponding Spearman correlation coefficients (r) are depicted inside each plot.

Supplementary Tables

Table S1: Age distribution of the sample sets analyzed by Sanger sequencing and SNaPshot methodologies.

Method / Age range	Sanger sequencing	SNaPshot
	**N**	**N**
1-19 years	16	13
20-29 years	16	10
30-39 years	13	13
40-49 years	24	22
50-59 years	39	37
60-69 years	27	22
70-79 years	30	30
80-89 years	17	19
90-94 years	3	2
1-94 years	185	168

Table S2: Univariate linear regression analysis of the 43 CpG sites in *ELOVL2, FHL2, EDARADD, PDE4C* and *C1orf132* loci in 185 samples including blood from living and deceased individuals, teeth from living and deceased individuals and bone collected during autopsies.

Gene	CpG site	Location	β	R	R^2	Corrected R^2	SE	*P*-value
ELOVL2	CpG1	Chr6: 11044628	136.914	0.739	0.547	0.544	15.19	**2.79×10^{-33}**
	CpG2	Chr6: 11044631	106.822	0.638	0.407	0.404	17.38	**1.58×10^{-22}**
	CpG3	Chr6: 11044634	211.972	0.693	0.481	0.478	16.26	**7.63×10^{-28}**
	CpG4	Chr6: 11044640	125.867	0.652	0.425	0.422	17.11	**9.53×10^{-24}**
	CpG5	Chr6: 11044642	114.293	0.740	0.547	0.545	15.18	**2.55×10^{-33}**
	**CpG6**	Chr6: 11044644	**188.014**	**0.759**	0.576	0.573	14.70	**6.87×10^{-36}**
	CpG7	Chr6: 11044647	228.823	0.663	0.440	0.436	16.89	**8.71×10^{-25}**
	CpG8	Chr6: 11044655	135.278	0.645	0.416	0.413	17.24	**3.74×10^{-23}**
	CpG9	Chr6: 11044661	140.696	0.615	0.378	0.374	17.80	**1.35×10^{-20}**
FHL2	**CpG1**	Chr2:105399282	**139.052**	**0.692**	0.479	0.476	16.29	**1.11×10^{-27}**
	CpG2	Chr2:105399288	127.199	0.635	0.404	0.400	17.43	**2.62×10^{-22}**
	CpG3	Chr2:105399291	133.508	0.640	0.410	0.407	17.33	**9.95×10^{-23}**
	CpG4	Chr2:105399297	184.205	0.523	0.273	0.269	19.24	**2.32×10^{-14}**
	CpG5	Chr2:105399300	158.887	0.647	0.419	0.416	17.02	**2.43×10^{-23}**
	CpG6	Chr2:105399310	158.657	0.538	0.290	0.286	19.01	**2.70×10^{-15}**
	CpG7	Chr2:105399314	96.186	0.308	0.095	0.090	21.47	**0.000020**
	CpG8	Chr2:105399316	112.689	0.389	0.151	0.147	20.79	**4.43×10^{-8}**
	CpG9	Chr2:105399323	25.114	0.088	0.008	0.002	22.48	0.235207
	CpG10	Chr2:105399327	5.881	0.020	0.000	-0.005	22.56	0.787338
	CpG11	Chr2:105399338	-125.337	-0.476	0.237	0.223	19.84	**7.34×10^{-12}**
	CpG12	Chr2:105399340	-35.520	-0.140	0.020	0.014	22.34	0.056883
EDARADD	CpG1	Chr1:236394458	1.873	0.007	0.000	-0.005	22.57	0.924703
	CpG2	Chr1:236394441	-109.086	-0.507	0.257	0.253	19.45	**1.85×10^{-13}**
	**CpG3**	Chr1:236394382	**-189.185**	**-0.682**	0.465	0.462	16.51	**1.21×10^{-26}**
	CpG4	Chr1:236394370	-101.037	-0.410	0.168	0.163	20.58	**6.95×10^{-9}**
PDE4C (N = 196)	CpG1	Chr19:18233139	96.130	0.502	0.252	0.248	19.52	**3.46×10^{-13}**
	**CpG2**	Chr19:18233133	**103.448**	**0.613**	0.376	0.372	17.83	**1.79×10^{-20}**
	CpG3	Chr19:18233131	97.447	0.559	0.313	0.309	18.71	**1.34×10^{-16}**
	CpG4	Chr19:18233127	45.141	0.238	0.057	0.051	21.92	**0.001120**
	CpG5	Chr19:18233105	100.544	0.462	0.213	0.209	20.02	**3.71×10^{-11}**
	CpG6	Chr19:18233091	-30.238	-0.157	0.025	0.019	22.29	**0.032991**
	CpG7	Chr19:18233082	10.797	0.048	0.002	-0.003	22.54	0.516987
	CpG8	Chr19:18233079	-44.121	-0.173	0.030	0.025	22.24	**0.018301**
	CpG9	Chr19:18233070	-77.591	-0.313	0.098	0.093	21.43	**0.000014**
	CpG10	Chr19:18233058	-15.015	-0.054	0.003	-0.003	22.56	0.471827
	CpG11	Chr19:18233048	-28.244	-0.099	0.010	0.004	22.57	0.186649
	CpG12	Chr19:18233042	-5.655	-0.021	0.000	-0.005	22.68	0.782908
C1orf132 (N = 126)	**CpG1**	Chr1:207823681	**-135.517**	**-0.654**	0.428	0.425	17.07	**5.67×10^{-24}**
	CpG2	Chr1:207823675	-97.150	-0.592	0.351	0.347	18.18	**6.62×10^{-19}**
	CpG3	Chr1:207823672	-88.567	-0.572	0.327	0.324	18.51	**1.79×10^{-17}**
	CpG4	Chr1:207823660	-89.102	-0.549	0.301	0.297	18.88	**7.38×10^{-16}**
	CpG5	Chr1:207823657	-96.852	-0.570	0.325	0.321	18.60	**4.49×10^{-17}**
	CpG6	Chr1:207823637	-76.285	-0.448	0.201	0.196	20.19	**1.78×10^{-10}**

Abbreviations: N, number of samples; β, unstandardized coefficient; R, correlation coefficient; SE, standard error. Significant p-values are in bold. The strongest age-

associated CpG site is in bold and underlined. Genomic positions were based on the GRCh38/hg38 assembly.

Table S3: Statistical parameters obtained in a multiple regression model with the seven CpGs in genes *ELOVL2, FHL2, EDARADD, PDE4C* and *C1orf132* selected by stepwise regression approach, in blood, bone and tooth samples.

**Marker**	**Coefficient**	***P*-value**
(Intercept)	26.852	0.009
EDARADD CpG3	-24.767	0.016
FHL2 CpG5	68.537	0.000
FHL2 CpG11	-51.319	0.000
ELOVL2 CpG5	57.461	0.000
PDE4C CpG5	41.449	0.000
PDE4C CpG9	-66.397	0.000
C1orf132 CpG3	-27.418	0.000

Table S4: Statistical parameters obtained in a multiple regression model with the three CpGs in genes *ELOVL2, C1orf132* and *KLF14*, selected by stepwise regression approach, in blood, bone and tooth samples.

**Marker**	**Coefficient**	***P*-value**
(Intercept)	29.220	0.000
C1orf132	-33.437	0.000
ELOVL2	96.850	0.000
KLF14	208.747	0.000
